# Delineating trajectories of alcohol consumption and alcohol problems from adolescence to young adulthood: An integrated assessment of genetic, familial, and psychosocial factors

**DOI:** 10.1111/acer.70166

**Published:** 2025-10-31

**Authors:** Hui G. Cheng, Jon Heron, Matthew Hickman, Alexis C. Edwards

**Affiliations:** ^1^ Virginia Commonwealth University Richmond Virginia USA; ^2^ Population Health Sciences University of Bristol Bristol UK; ^3^ Department of Psychiatry, Virginia Institute for Psychiatric and Behavioral Genetics Virginia Commonwealth University Richmond Virginia USA

**Keywords:** adolescence, ALSPAC, AUDIT, risk factors, trajectory of drinking

## Abstract

**Background:**

Few studies have jointly assessed the relationships of genetic, environmental, and psychosocial factors with the trajectory of alcohol consumption and alcohol problems over time. In this study, we estimate relationships between a host of predictors (measured before age 16 years) and the trajectories of alcohol consumption and alcohol problems measured at five different time points, spanning from mid‐adolescence (16 years) to early adulthood (23 years) using data from a large population‐based birth cohort from the United Kingdom (UK).

**Methods:**

The Alcohol Use Disorders Identification Test (AUDIT) was used to assess alcohol consumption and problematic drinking at approximately ages 16, 18, 19, 21, and 23 years among participants of the Avon Longitudinal Study of Parents and Children (ALSPAC). Predictors (measured before age 16) included polygenic risk scores derived based on results from the UK Biobank, family history of drinking problems, parental monitoring, indicators of internalizing and externalizing problems, smoking, and personality measures. Latent growth models were used for analysis.

**Results:**

Results from growth models showed that most variables, including polygenic risk scores, were associated with the initial stage (i.e., the intercept), while a few variables were associated with the rate of change (i.e., slopes), such as being a female, family history of alcohol problems, and peer group deviance.

**Conclusions:**

Findings from this study indicate that genetic, familial, and personality traits related to externalization were associated with the initial level of drinking or drinking‐related problems, whereas fewer variables were associated with the change in drinking or drinking‐related problems over time. These findings suggest that these variables can be used to identify high‐risk individuals for drinking problems early on, and it is necessary to consider age or developmental stage in alcohol research.

## Graphical abstract

This study used data from the UK‐based ALSPAC cohort to examine how genetic, environmental, and psychosocial factors measured before age 16 predict trajectories of alcohol consumption and problems from ages 16 to 23. Latent growth models showed most predictors were linked to initial levels, while fewer predicted change over time. Findings highlight early identification of high‐risk youth and the importance of developmental context.

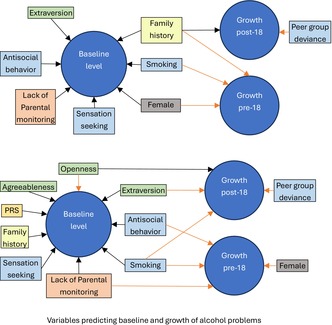

## INTRODUCTION

Alcohol consumption and drinking‐related problems are major contributors to premature morbidity and mortality (Whiteford et al., 2013). Knowledge on how genetic, environmental, and psychosocial factors precipitate these complex phenotypes can provide useful information on the design of prevention strategies.

Adolescence to young adulthood is the key period when alcohol consumption initiates and increases, and alcohol problems first emerge (Cheng, Cantave, & Anthony, 2016; Grant et al., 2001; Pedersen & Skrondal, 1998). Numerous studies have documented dynamic changes of genetic and environmental influences on drinking‐related outcomes during this developmental period. For example, a landmark twin study documented that common environmental factors played a key role in alcohol consumption before early adolescence and declined afterwards. In contrast, genetic influences started to increase in early adolescence until early adulthood; across all ages, unique environment accounted for the largest portion of variances (Kendler et al., 2008). Further analysis revealed that genetic influences during adolescence and adulthood may be qualitatively different (Edwards & Kendler, 2013). In an analysis that dissected the genetic factor into an alcohol‐specific genetic factor and a general externalizing (i.e., nonalcohol‐specific) genetic factor, differential trajectories were found at different ages—although the influence of both factors rapidly rose during adolescence and peaked during mid‐adolescence, the influence of the nonalcohol‐specific factor declined afterwards, whereas the influence of the alcohol‐specific genetic factor plateaued afterwards (Kendler et al., 2011).

Evidence about the relative contribution, within an integrative model, of multiple genetic, environmental, and psychosocial factors on the trajectory of alcohol consumption and alcohol problems over time is scarce. Previous studies have investigated the prediction of alcohol‐related outcomes using a few different approaches. One focused on the architecture of a broad range of psychosocial variables measured from childhood to late adolescence (i.e., as early as 4 years and up to 17.5 years) predicting the level of alcohol problems at age 20 (Edwards et al., 2016). Findings suggested (1) robust predictive pathways cascading from early externalizing; and (2) measures more proximal to problematic drinking at age 20 were more robust predictors compared with more distal measures even though the cascade can begin as early as age 11 or 12. This shed light on the dynamic nature of drinking problems during the transition from late adolescence to early adulthood and accentuated the need to adopt a developmental perspective toward drinking‐related outcomes.

In the current study, we sought to estimate relationships between genetic, familial, and sociopsychological variables (measured before age 16 years) and the trajectory of alcohol consumption and alcohol problems measured at five different age points spanning from mid‐adolescence (16 years) to early adulthood (23 years) using data from a large birth cohort from the United Kingdom (UK). Building upon previous studies on overall Alcohol Use Disorder Identification Test (AUDIT) scores (Edwards et al., 2017), we model an alcohol consumption score and an alcohol problem score separately to probe for potential similarities and variations in prediction structures between these two correlated but at least partially distinct phenotypes (Babor & Grant, 1989; Kranzler et al., 2019; Mallard et al., 2022; Olsson et al., 2016; Sanchez‐Roige et al., 2019; Wennberg et al., 2000). Leveraging advances in the estimation of genetic risks of health conditions, including alcohol consumption and problematic drinking, through the availability of large‐scale genome‐wide association studies during the past decade (Johnson et al., 2020), we included polygenic scores for alcohol consumption and alcohol problems generated using independent data sources as predictors. Our selection of predictors was guided by developmental models of substance use, particularly biopsychosocial frameworks emphasizing the interaction between biological, psychological, and environmental influences on risk for alcohol use behaviors (Dick et al., 2014; Meyers et al., 2014). Specifically, we selected variables that represent major risk domains identified in prior research: genetic predisposition (polygenic risk scores and family history of drinking problems), personality traits (e.g., the Big Five and sensation seeking), internalizing symptoms (depressive symptoms), smoking behavior, and social‐contextual factors, such as parental monitoring and peer affiliation, two of the most important social factors for drinking behaviors during adolescence and early adulthood (Stephenson et al., 2025). Using a growth model approach, we investigated whether there are differential associations for the baseline level and the growth over time.

## METHODS

### Study sample

The Avon Longitudinal Study of Parents and Children (ALSPAC) is a longitudinal, population‐based cohort study of parents and children from southwest England (Boyd et al., 2013; Fraser et al., 2013; Northstone et al., 2019). Pregnant women residing in the southwest of England who had an estimated delivery date between April 1991 and December 1992 were invited to participate. The initial study cohort consisted of 14,541 pregnancies and 14,901 singletons and twins (52% boys and 48% girls) still alive at 12 months of age. The study website contains detailed information on the data, which is available through a fully searchable data dictionary and variable search tool (AVON Study Team, 2024). Ethics approval for the study was obtained from the ALSPAC Ethics and Law Committee and the Local Research Ethics Committees. Informed consent for the use of data collected via questionnaires and clinics was obtained from participants following the recommendations of the ALSPAC Ethics and Law Committee at the time. Consent for biological samples has been collected in accordance with the Human Tissue Act (2004).

### Assessment

#### AUDIT assessment

The AUDIT was used to assess alcohol consumption and problematic drinking at approximately ages 16, 18, 19, 21, and 23 years to all participants (Babor et al., 2001). The assessments at age 18 were self‐administered electronically within a clinic. The assessments at other time points were self‐administered via postal or online questionnaire. The AUDIT score for alcohol consumption (AUDIT‐C) was calculated by summing the first three AUDIT items, and the AUDIT score for problematic drinking was calculated by summing the last seven items (AUDIT‐P). Higher scores represent higher levels of consumption or problematic drinking, respectively. Participants who had never consumed alcohol were excluded from the current study.

#### Genotyping

Genotypes for ALSPAC participants are available for a fee to researchers with an approved project (see http://www.bristol.ac.uk/alspac/researchers/ for details). Genotyping and initial quality control of data were performed by ALSPAC analysts, unrelated to the current project. Genotyping in ALSPAC was performed on the Illumina HumanHap550 quad genome‐wide SNP genotyping platform by 23andMe subcontracting the Wellcome Trust Sanger Institute, Cambridge, UK, and the Laboratory Corporation of America, Burlington, NC, USA. Individuals were excluded from analyses on the basis of excessive or minimal heterozygosity, sex mismatch, individual missingness (0.3%), cryptic relatedness as measured by identity by descent (genome‐wide IBD 0.10%) and sample duplication. Individuals were assessed for population stratification using multidimensional scaling modeling seeded with HapMap Phase II release 22 reference populations. Individuals of non‐European ancestry were removed from further analysis. ShapeIt v2 was used to impute to 1000 Genomes Phase 1, Version 3, Release December 2013. We excluded markers with MAF <0.01, deviation from HWE (*p* < 5 × 10^−6^), genotyping rate <0.95, or INFO <0.80.

#### Polygenic risk scores

Polygenic risk scores (PRS) were derived using GWAS results from UK Biobank and 23andMe (Sanchez‐Roige et al., 2019). PRS‐CS (Ge et al., 2019), which relies on a Bayesian regression framework and performs a continuous shrinkage prior to the effect sizes of SNPs, was used to derive appropriate SNP weights. The 1000 Genomes European reference panel was used given the ancestry profile of ALSPAC participants. With the resulting SNP weights, polygenic risk scores were derived using the ‐‐score and ‐‐dosage options in Plink 1.9 (www.cog‐genomics.org/plink/1.9/).

#### Psychosocial and behavioral variables

Multiple demographic and psychosocial variables were included in this analysis, including sex, ancestry principal component scores, family history of alcohol problems, conduct problems or antisocial behavior (at 15 years and 6 months, 15y6m), lack of parental monitoring (15y6m), symptoms of depression (at 13y6m), lifetime cigarette consumption (at 15y6m), sensation seeking behavior (13y6m), personality traits (extraversion, agreeableness, conscientiousness, openness, and neuroticism at 13y6m), and peer group deviance (12y6m). Some of these variables were measured at multiple time points. In this analysis, the measure taken immediately before age 16 was used. The supplementary material Data S1 and a previous publication provide more details about these covariates (Edwards et al., 2016). Study data were collected and managed using Research Electronic Data Capture (REDCap) electronic data capture tools hosted at the University of Bristol (Harris et al., 2009). REDCap is a secure, web‐based software platform designed to support data capture for research studies.

### Analysis

**FIGURE 1:**
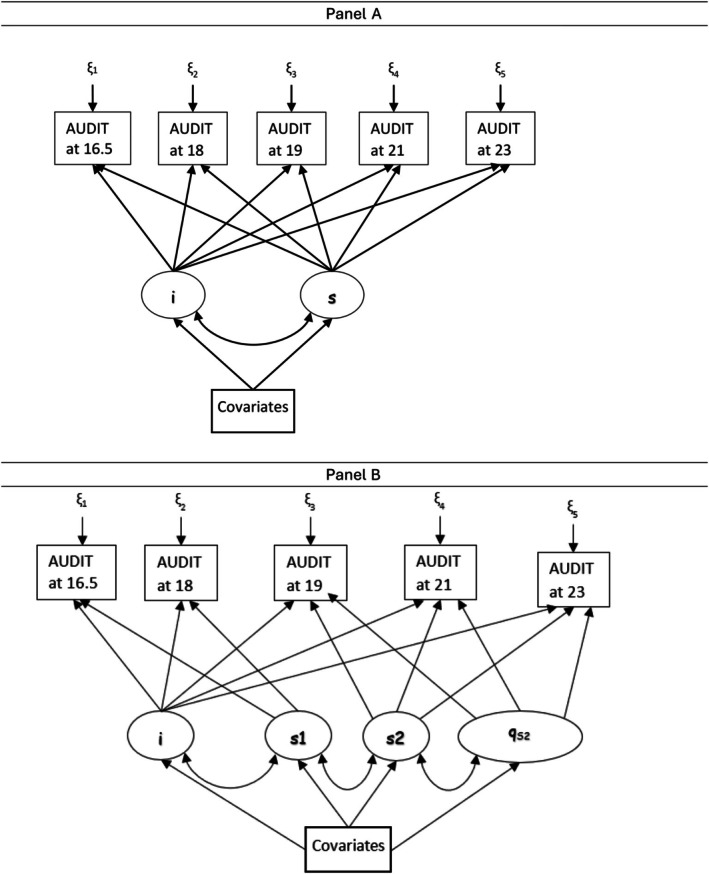
Depiction of latent growth models. Panel A: A simple growth model with an intercept and a slope. Panel B: Growth model with an intercept, two slopes, and a quadratic growth with the second slope. Labels for latent variables: I—intercept; q_s2_—quadratic slope after age 18; s1—slope before age 18; s2—slope after age 18.

In this study, we used a latent variable growth model approach to assess how genetic, familial, and psychosocial variables under study were associated with the initial status (i.e., intercept) or the growth over time (i.e., slope) for AUDIT‐C and AUDIT‐P. Panel A of Figure 1 provides a depiction of the conceptual model. In this model, the intercept represents the initial state (outcome variable at the first time point), and the slope represents the change in the outcome variable for a one‐unit increase in the time score (Duncan & Duncan, 2009). Jointly, the intercept and slope(s) define the trajectory of development over time. In this study, we specified separate growth rates (i.e., slopes) before and after age 18 years because (1) the legal age to purchase alcoholic beverages in the UK is 18; drinking behavior and context may change once an individual can legally purchase alcoholic drinks on their own; and (2) many individuals gain incremental independence around age 18 as a result of entry to college or the workforce. We first fit growth models without covariates to assess the goodness of fit of these models. In this study, a quadratic growth parameter was added to allow for the tapering down after age 21 (Figure 1 Panel B). In the next steps, we regressed all variables (including 10 ancestry informative genetic principal component scores to account for potential population stratification) on the intercept and the slopes to estimate the relationships between these covariates and the latent intercepts and slopes. We used multiple indices to assess the goodness of fit, including Root Mean Square Error of Approximation (RMSEA) (Steiger, 1990), comparative fit index (CFI) (Bentler, 1990), and Tucker–Lewis Index (TLI) (Tucker & Lewis, 1973). An RMSEA < 0.05 and CFI/TLI >0.95 are considered indications of generally good fit (Bentler & Bonett, 1980; Hu & Bentler, 1999). We specified maximum likelihood with robust errors estimator for growth models. In addition to growth models, we used random‐intercept models to estimate the association between antecedent sociopsychological variables and AUDIT‐C and AUDIT‐P scores at each time point. Stata (version MP 18.0; StataCorp, 2022) and Mplus (version 8.9; Muthén & Muthén, 1998–2023) were used for data analysis. The analysis was not preregistered, and the results should be considered exploratory.

## RESULTS

**FIGURE 2:**
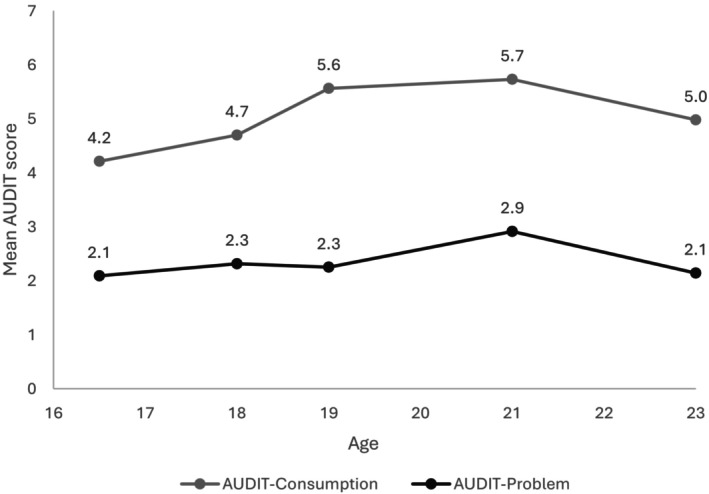
Estimated mean AUDIT scores. Data from ALSPAC.

Information about AUDIT was available for 7279 participants (57% male) at one or more of the five follow‐ups. Compared with the full ALSPAC cohort, participants in the analytic sample were more likely to be male (57% vs. 49%) and to have mothers with education beyond high school (48% vs. 28%). They were less likely to have parents who owned their home (16% vs. 27%). Because the analysis included only ever drinkers, these differences may partly reflect characteristics that distinguish ever from never drinkers. The groups were similar in the prevalence of a family history of drinking problems (9% in both). Because sociopsychological variables were measured at different time points, there were varying amounts of missing data ranging from 3322 for parental monitoring to 0 for sex or PRS. Participants who did and did not have missing values in variables studied are similar in AUDIT‐C and AUDIT‐P PRS (*p* = 0.602 and 0.483, respectively). They were also similar with respect to family history of alcohol problems (9.2% vs. 8.9% *p* = 0.670). Those without missing values were slightly less likely to be male compared to those with any missing values in any of the covariates (55% vs. 58%, *p* = 0.041). Those without missing values had higher levels of maternal education (51% vs. 43% with ≥high school maternal education, *p* < 0.001), and they were less likely to have parents who owned their home (12% vs. 20%, *p* < 0.001). Mean AUDIT scores at each age/follow‐up are shown in Figure 2. The pattern of the mean AUDIT consumption score over time can be characterized as incremental increases from 16 to 19 years of age, followed by a plateau between 19 and 21 and a decrease between 21 and 23. AUDIT problem score increased slightly between 16 and 18, peaked at 21, and returned to the level at 16 at 23.

For alcohol consumption, the growth model depicted in Figure 1 Panel B fit the data reasonably well (RMSEA = 0.056, 90% CI = 0.048 to 0.065; CFI = 0.977; TLI = 0.955; SRMR = 0.022). The growth curve was characterized by a smaller growth between 16 and 18, a greater growth after age 18 with a negative quadratic growth representing a reversal after the peak around age 21. Next, we regressed sociopsychological variables on the intercept and slopes. As shown in Table 1 (and Table S2 for standardized estimates), the PRS for alcohol consumption, family history of alcohol problems, sensation seeking, and agreeableness were positively associated with the intercept; openness was inversely associated with the intercept. A lack of parental monitoring, antisocial behavior, and lifetime cigarette consumption were positively associated with the intercept and inversely associated with the slope (i.e., slower growth) before 18. Extraversion was positively associated with the intercept and inversely associated with the growth after 18 (i.e., slower growth); the opposite was observed for openness. For variables associated with both intercept and growth, the directionality of association was opposite for the intercept and growth parameter. Being a female was not associated with the intercept but was associated with slower growth of alcohol consumption before 18. The observed patterns are consistent with results from random‐intercept models (Table S4).

**TABLE 1:** Estimated association linking genetic and psychosocial variables with the intercept and growth of AUDIT *alcohol consumption* subscale score.

	Intercept = 4.15	Slope 1 = 0.45	Slope 2 = 1.04	Quadratic growth = −0.18
Female	−0.13 (−0.37, 0.10)	**−0.29 (−0.44, −0.13)**	0.17 (−0.02, 0.37)	−0.04 (−0.08, 0.00)
Polygenic score for alcohol consumption	**0.22 (0.13, 0.32)**	−0.05 (−0.11, 0.02)	0.05 (−0.03, 0.13)	−0.01 (−0.02, 0.01)
Family history of alcohol problems	**0.39 (0.05, 0.72)**	0.00 (−0.23, 0.22)	0.19 (−0.10, 0.48)	−0.05 (−0.10, 0.01)
Lack of parental monitoring	**0.18 (0.07, 0.29)**	**−0.07 (−0.15, −0.001)**	0.02 (−0.07, 0.10)	−0.01 (−0.02, 0.01)
Sensation seeking	**0.37 (0.24, 0.49)**	−0.04 (−0.12, 0.05)	0.02 (−0.08, 0.12)	0.00 (−0.02, 0.02)
Antisocial behavior	**0.51 (0.37, 0.65)**	**−0.10 (−0.20, −0.001)**	−0.01 (−0.12, 0.10)	0.00 (−0.02, 0.02)
Peer group deviance	−0.02 (−0.14, 0.11)	−0.03 (−0.11, 0.06)	**−0.10 (−0.21, −0.01)**	**0.02 (0.002, 0.04)**
Neuroticism	−0.01 (−0.14, 0.12)	0.04 (−0.05, 0.12)	0.02 (−0.08, 0.12)	0.00 (−0.02, 0.02)
Extraversion	**0.27 (0.16, 0.38)**	0.06 (−0.01, 0.13)	**−0.09 (−0.18, −0.01)**	0.01 (−0.01, 0.03)
Openness	**−0.16 (−0.27, −0.04)**	0.03 (−0.05, 0.11)	**0.10 (0.003, 0.19)**	−0.02 (−0.04, 0.001)
Agreeableness	**0.15 (0.04, 0.27)**	0.01 (−0.07, 0.09)	−0.02 (−0.12, 0.08)	0.00 (−0.02, 0.02)
Conscientiousness	−0.10 (−0.21, 0.02)	−0.05 (−0.12, 0.03)	−0.02 (−0.11, 0.07)	0.01 (−0.01, 0.02)
Depressive symptoms	−0.02 (−0.15, 0.11)	−0.02 (−0.11, 0.07)	−0.07 (−0.18, 0.04)	0.01 (−0.01, 0.03)
Lifetime cigarette consumption	**0.41 (0.33, 0.49)**	**−0.06 (−0.12, −0.001)**	**−0.18 (−0.25, −0.11)**	**0.03 (0.01, 0.04)**

*Note*: Data from the Avon Longitudinal Study of Parents and Children Study 1991–2014. RMSEA = 0.041 (90% CI = 0.033 to 0.048); CFI = 0.977; TLI = 0.902. Slope 1 represents the growth from age 16.5 to age 18. Slope 2 and quadratic growth represent the growth from age 18 to age 23. Bold font indicates statistical significance at 0.05 level.

For problematic drinking, the growth model depicted in Figure 1 Panel B fit the data reasonably well (RMSEA = 0.034, 90% CI = 0.026 to 0.043; CFI = 0.975; TLI = 0.959; SRMR = 0.030). As shown in Table 2 (and Table S3 for standardized estimates), multiple variables were associated with the intercept, including a lack of parental monitoring, antisocial behaviors, sensation seeking, extraversion, and lifetime cigarette consumption. Family history was positively associated with the intercept and inversely associated with the slope (i.e., slower growth) before 18, and positively associated with the slope (i.e., faster growth) after 18. Females had a higher level of drinking problems at age 16 (i.e., positive association with the intercept) but had a slower rate of growth compared with males. Participants who smoked more cigarettes also had higher levels of drinking problems at age 16 but slower growth between 16 and 18. Of note, the PRS for problematic drinking was not associated with any of the parameters. The observed patterns are consistent with results from random‐intercept models (Table S5). In exploratory models, we included both PRS for alcohol consumption and problematic drinking in the growth models in order to explore the potential role of one PRS while holding the other constant. The PRS for alcohol problems was not associated with any of the parameters in the alcohol consumption model, and there were minimal changes in estimates of other variables. The PRS for alcohol consumption was positively associated with the intercept of the problematic drinking model (coef. = 0.13; 95% CI = 0.01, 0.25; *p* = 0.038), and there were minimal changes in estimates of other variables. Of note, the PRS for problematic drinking was not associated with any parameters of the growth model.

**TABLE 2:** Estimated association linking genetic and psychosocial variables with the intercept and growth of AUDIT alcohol problem subscale score.

	Intercept = 1.47	Slope 1 = 0.44	Slope 2 = 0.57	Quadratic growth = −0.09
Female	**0.61 (0.36, 0.87)**	**−0.47 (−0.66, −0.27)**	−0.20 (−0.45, 0.05)	0.02 (−0.03, 0.07)
Polygenic score for alcohol problems	0.09 (−0.02, 0.19)	0.06 (−0.02, 0.14)	−0.02 (−0.12, 0.08)	0.01 (−0.01, 0.02)
Family history of alcohol problems	**0.85 (0.36, 1.33)**	**−0.42 (−0.73, −0.11)**	**0.57 (0.18, 0.96)**	**−0.10 (−0.17, −0.03)**
Lack of parental monitoring	**0.22 (0.09, 0.36)**	−0.08 (−0.17, 0.02)	0.04 (−0.07, 0.14)	−0.01 (−0.03, 0.01)
Sensation seeking	**0.18 (0.05, 0.32)**	−0.09 (−0.20, 0.01)	0.06 (−0.07, 0.18)	−0.01 (−0.03, 0.02)
Antisocial behavior	**0.62 (0.44, 0.79)**	−0.02 (−0.15, 0.10)	−0.09 (−0.26, 0.08)	0.02 (−0.01, 0.05)
Peer group deviance	0.01 (−0.16, 0.19)	0.12 (−0.01, 0.24)	**−0.17 (−0.31, −0.04)**	**0.03 (0.01, 0.06)**
Neuroticism	0.01 (−0.14, 0.16)	0.04 (−0.06, 0.14)	0.05 (−0.08, 0.18)	−0.01 (−0.03, 0.01)
Extraversion	**0.15 (0.02, 0.27)**	0.06 (−0.03, 0.15)	0.04 (−0.07, 0.15)	−0.01 (−0.03, 0.01)
Openness	−0.03 (−0.15, 0.09)	−0.01 (−0.10, 0.09)	0.05 (−0.07, 0.17)	−0.01 (−0.03, 0.01)
Agreeableness	0.10 (−0.03, 0.23)	0.02 (−0.09, 0.12)	0.01 (−0.12, 0.14)	0.00 (−0.03, 0.02)
Conscientiousness	−0.04 (−0.18, 0.10)	−0.05 (−0.14, 0.04)	−0.10 (−0.21, 0.02)	0.02 (−0.00, 0.04)
Depressive symptoms	0.15 (−0.01, 0.31)	−0.02 (−0.13, 0.09)	0.06 (−0.07, 0.20)	−0.02 (−0.04, 0.01)
Lifetime cigarette consumption	**0.33 (0.23, 0.44)**	**−0.09 (−0.17, −0.01)**	−0.03 (−0.13, 0.07)	0.00 (−0.02, 0.02)

*Note*: Data from the Avon Longitudinal Study of Parents and Children Study 1991–2014. RMSEA = 0.028 (90% CI = 0.021 to 0.036); CFI = 0.982; TLI = 0.927. Slope 1 represents the growth from age 16.5 to age 18. Slope 2 and quadratic growth represent the growth from age 18 to age 23. Bold font indicates statistical significance at 0.05 level.

## DISCUSSION

In this study, we assessed prospective relationships between genetic, demographic, and sociopsychological variables and trajectories of two related but partially distinct drinking‐related phenotypes—alcohol consumption and drinking‐related problems—using data from a large population‐based longitudinal study in the UK. By using a latent growth model approach, we were able to model the initial stage (i.e., intercept) and growth over time simultaneously and test the association between key exposure variables during childhood and adolescence and whether these varied between alcohol consumption and problem drinking (Muthén & Curran, 1997). In addition, we used PRS derived from a large independent meta‐analysis of measurements of the same phenotypes (Sanchez‐Roige et al., 2019), which helped minimize the potential impact of sampling errors associated with a single sample. Findings from this study indicate that genetic, familial, and psychosocial factors were all prospectively associated with alcohol consumption and drinking problems over time, with some notable differences across outcomes discussed further below.

We found that both PRS and family history were associated with alcohol‐related phenotypes after adjusting for each other and an array of sociopsychological variables, which suggests that family history and PRS may represent unique characteristics in familial influences on alcohol‐related phenotypes (Wang et al., 2023), consistent with recent findings from other psychiatric contexts (Dybdahl Krebs et al., 2023). We observed stronger associations for family history, which may reflect risks conferred by environmental factors within the family. This finding accentuates the role of family environment in shaping an individual's drinking behavior early on and echoes the notion that PRS provides useful information and can be used to predict behavioral phenotypes along with phenotypic variables (Agerbo et al., 2021; Wang et al., 2023).

In this study, we found that *PRS for alcohol consumption* derived from genome‐wide association studies was associated with intercepts (the initial stage) but not changes over time for both alcohol consumption and drinking problems, extending findings from a previous study showing cross‐sectional associations between PRS and AUDIT phenotypes (Johnson et al., 2020). It is not immediately clear why the PRS was only associated with the intercept but not changes over time. It is possible that different sets of genes are associated with the initial stage and changes over time. For example, a previous study found that GABA2 gene polymorphisms were associated with change in alcohol drinking between age 18 and 19 years but not with the initial level at age 14 (Dick et al., 2014). Of note, the PRS used in the current analyses was based on GWAS of adult drinkers using cross‐sectional data, which might encompass a mixture of genes related to the initial stage and change over time when individuals at different ages were pooled together. Future studies on PRS using longitudinal data will shed more light on this issue. It is interesting that the *PRS for drinking problems* was not associated with any parameters of either outcome, which suggests that the PRS for alcohol consumption may be a better predictor for alcohol‐related outcomes.

With respect to family history, we found that individuals with a family history of alcohol problems had a higher level of drinking‐related problems initially, a slower growth during late adolescence, and more rapid growth during young adulthood. In line with previous observations, we found that familial and genetic components of alcohol problem change over time (Edwards & Kendler, 2013; Kendler et al., 2008). This finding highlights the need to account for the dynamic nature of alcohol use and problems in genetics research, as different familial or genetic factors may account for the initial state versus change over time.

An interesting observation is that although girls did not have higher alcohol consumption either at the initial level or the change over time compared with boys, they had a higher initial level of alcohol problems at 16, which grew more slowly before age 18. After 18, there were no male–female differences. This is consistent with recent findings of a female excess in drinking and drinking‐related problems (e.g., heavy drinking episodes and alcohol dependence) during adolescence, especially early adolescence, which reverses in early adulthood (Cheng & Anthony, 2016, 2017, 2018a, 2018b; Cheng, Chandra, et al., 2016). Openness and agreeableness scores were associated with consumption but not problematic drinking. These observations support the notion that, while correlated, alcohol consumption and problematic drinking may be at least partially different phenotypes (Kranzler et al., 2019). These findings suggest that psychosocial predictors (such as a lack of parental monitoring, sensation seeking, antisocial behavior, extraversion, and cigarette smoking) can be used to identify high‐risk individuals early on, and proper prevention and intervention strategies can be applied to reduce the risk of developing drinking problems. These prevention and intervention strategies can range from low‐intensity monitoring to high‐intensity interventions delivered by teachers, parents, and peers depending on the severity of drinking problems and resources available.

Antisocial behavior was among the strongest predictors for both alcohol consumption and problematic drinking. Being a female, having a family history of drinking problems, and cigarette smoking were also strong predictors of problematic drinking at both the initial level and growth over time. For these variables, the directionality of association for the initial level and growth was reversed—positive association with the initial level and slower growth between 16 and 18 years of age. One possible explanation is that girls, adolescents who smoked cigarettes, and those with a family history of drinking problems might have an earlier onset of drinking problems (i.e., before 16 years of age) (Cheng & Anthony, 2016, 2018b). To a lesser extent, this pattern is also seen for antisocial behaviors. Future studies with assessments before age 16 will shed light on this issue.

Findings from this study should be interpreted with the following limitations in mind. First, the study participants were predominantly non‐Hispanic white from the UK. Future studies in other populations with different drinking cultures are needed to assess consistencies in findings across populations. Furthermore, genetic findings on alcohol‐related outcomes from ancestrally diverse samples remain somewhat limited; additional research is necessary to extend our understanding of whether aggregate genetic liability and psychosocial factors jointly impact the onset and persistence of alcohol consumption and problems in such samples. In this study, we derived the PRS based on the UK Biobank and 23andMe summary statistics. Advantages of using this approach include (1) cross‐ancestry polygenic scores typically explain less of the variance than within‐ancestry scores (Mester et al., 2023), and (2) both UK Biobank and 23andMe included AUDIT in the assessment, the main outcomes in this study. While larger and more diverse alcohol‐related GWAS exist (Deak et al., 2022; Zhou et al., 2023), the phenotype assessment is different from the AUDIT, which may introduce measurement‐related variations to estimates. Second, we included genetic, sociodemographic, behavioral, and psychological variables in this study, but they are not exhaustive. In this study, we focused on antecedent predictors measurable in childhood or early adolescence to better capture early risk processes. While other variables, such as age of alcohol use onset are known strong predictors of later alcohol trajectories (Stephenson et al., 2025), they are correlated with the trajectories of alcohol drinking in a more complex way and they may reflect early manifestations of the same underlying risk pathways influencing alcohol use and thus would complicate interpretations of independent predictive effects (Deak et al., 2021). Additionally, future studies incorporating PRS of other psychosocial variables may provide insights on the potential role of PRS‐ and non‐PRS‐related factors in the development of alcohol consumption and problems. Third, there were a significant number of missing values in some of the covariates. The full information maximum likelihood assumes missing at random, which cannot be fully assessed. Future studies with minimum attrition will provide further evidence about the potential impact of missing values. Fourth, although the majority (5 of 7) of AUDIT‐P items assess alcohol‐related problems in the past year, two items (injury and concern from others) allow for reporting of lifetime experiences. As a result, AUDIT‐P scores may reflect a mix of recent and past problems. Nonetheless, the total score is primarily geared toward capturing recent alcohol‐related problems, particularly given the relatively young age of the sample. Finally, this study assessed the overall trajectories of drinking‐related outcomes for the population as a whole. It is well possible that heterogeneous trajectories exist. Future studies on this issue will provide more nuanced insights about the nature of potential heterogeneities and subgroups of individuals who are more likely to follow different trajectories as they traverse through late adolescence and young adulthood, during which multiple competing causal mechanisms for drinking and related problems are at play.

## FUNDING INFORMATION

The UK Medical Research Council and Wellcome (grant ref.: 217065/Z/19/Z) and the University of Bristol provide core support for ALSPAC. This publication is the work of the authors and HGC and ACE will serve as guarantors for the contents of this paper. A comprehensive list of grants funding is available on the ALSPAC website (http://www.bristol.ac.uk/alspac/external/documents/grant‐acknowledgements.pdf). This research was specifically funded by NIH R01 AA018333.

## CONFLICT OF INTEREST STATEMENT

The authors declare no conflict of interest relevant to this study.

## Supporting information


Data S1

## Data Availability

The data that support the findings of this study are available from ALSPAC. Restrictions apply to the availability of these data, which were used under license for this study. Data are available from the author(s) with the permission of ALSPAC.
