# Supplementary material for: Delineating trajectories of alcohol consumption and alcohol problems from adolescence to young adulthood: An integrated assessment of genetic, familial, and psychosocial factors
Source: Alcohol Clin Exp Res (Hoboken). 2025 Oct 31;49(11):2527–36. doi: 10.1111/acer.70166 (PMC12638281; doi:10.1111/acer.70166)
Supplement: Supplementary file 1 — Data S1 [file ACER-49-2527-s001.docx]

**Supplemental Material**

**Approach to scoring individual variables -** The individual scores for the variables in this model were all sum scores from several individual items (except sex). If the proband provided non-missing responses to at least half the items for a particular variable, a prorated score was calculated. This was based on the mean response for the non-missing items and rescaled to the expected value if all items had non-missing values. Exceptions to this are noted below. Once raw scores were calculated variables were checked to see if transformations were necessary to reduce skewness resulting in overly influential observations. In order to do this we checked the skewness of the raw scores. If skewness was less than 1.0 the variable was considered sufficiently symmetric to use as is. If skewness was greater than 1.0, then a square root transformation was applied. If this brought skewness to be less than 1.0 the transformation was considered adequate. If not, variables were transformed using the natural logarithm which was sufficient to reduce skewness to less than 1 for almost all variables. The direction of scoring of all variables was in the presumed direction of risk for alcohol use/problems. All variables were converted to z-scores so that all were on a similar metric and to minimizing scaling issues.

**Choice of variables** - When multiple measures of the same trait at various ages were available, the one that was taken immediately before age 16 years was used.

**Variables**

**Sex** – The sex of child was coded as 0=female and 1=male. Available for all participants.

**Mother alcohol problems** - Based on 13 items about the existence of alcohol problems or alcoholism from both mother self-reports and partner reports on mother at various time intervals starting with prior to pregnancy up to 12 years 1 month. Individual items were converted to z-scores and if there were at least 4 non-missing items the mean of the non-missing items was considered a valid score. To compensate for the skewed nature of the variable, a constant was added to make the minimum score equal to 1 and transformed using the natural logarithm. Available for 5,026 participants.

**Peer Group Deviance 12y6m** - The score for this item involved 12 binary items about various anti-social behaviors in the adolescent’s peer group. The item on fighting was divided into two items. One was scored positive for fighting of any kind while the second was scored positive if the fighting caused injury or involved use of a weapon. This resulted in 13 items which were summed. As long as at least 7 of the 13 items were non-missing a prorated score was developed. This score was transformed using the natural logarithm and converted to a z-score. Available for 4,083 participants.

**Extraversion 13y6m** - Based on ten items from the International Personality Item Pool (Ehrhart *et al.*, 2008). If at least five items were non-missing a pro-rated score was developed which was converted to a z-score. Available for 3,900 participants.

**Conscientiousness 13y6m** - Based on ten items from the International Personality Item Pool. If at least five items were non-missing a pro-rated score was developed which was converted to a z-score. This item was reverse coded to represent lack of conscientiousness. Available for 3,901 participants.

**Openness 13y6m** - Based on ten items from the International Personality Item Pool. If at least five items were non-missing a pro-rated score was developed which was converted to a z-score. This item was reverse coded to represent lack of conscientiousness. Available for 3,899 participants.

**Agreeableness 13y6m** - Based on ten items from the International Personality Item Pool. If at least five items were non-missing a pro-rated score was developed which was converted to a z-score. This item was reverse coded to represent lack of conscientiousness. Available for 3,898 participants.

**Neuroticism 13y6m** - Based on ten items from the International Personality Item Pool. If at least five items were non-missing a pro-rated score was developed which was converted to a z-score. This item was reverse coded to represent lack of conscientiousness. Available for 3,901 participants.

**Sensation Seeking 13y6m -** This score was based on 21 items with 4 ordinal levels from a modified version of Arnett’s Inventory of Sensation Seeking (Arnett, 1994). As long as there were at least 11 non-missing items a pro-rated score was developed ad converted t o a z-score. Available for 3,885 participants.

**Lack of Parental Monitoring 15y6m** - Parental monitoring was measured with 24 items with 5 levels of ordinal response. Six of these items were based on peer group and were not scored for adolescents without a peer group. An additional six items concerned school and were not scored for those who did not attend school. Thus most adolescents had 24 items in this scale, but some may have had 18 or 12 items. As long as at least half of the scored items had non-missing responses a pro-rated score was calculated. The pro-rated score was converted to a z-score. Available for 3,324 participants.

**Conduct Disorder 15y6m** - Based on a list of 22 types of delinquent or anti-social behavior in the past year. Scoring was 0 for never, 1 for once, 2 for 2-5 times, and 3 for 6 or more times. As long as there were at least 11 non-missing responses, the score was pro-rated. The skewed sum score was transformed using the natural logarithm and converted to a z-score. Available for 3,639 participants.

**Major Depression 16y6m -** Based on the Short Mood and Feelings Questionnaire (Angold *et al.*, 1995), a 13 item list with 3 levels per item of depression symptoms. A pro-rated scale was created as long as at least 7 items were non-missing. The score was transformed using the square root and converted to a z-score. Available for 3,907 participants.

**Lifetime cigarette consumption 15y6m** – Based on participants’ self-reported cigarette consumption. The variable has 6 categories representing 0 (i.e., never smoked), <5, 5-19, 20-49, 50-99 and 100+ cigarettes. Available for 3,585 participants.

| **Table S1. Sample sizes at each wave for each variable studied.** | | | | | |
| --- | --- | --- | --- | --- | --- |
| **Follow-up age** | **16** | **18** | **19** | **21** | **23** |
| **Overall** | 5035 | 4183 | 3343 | 4178 | 3910 |
| Sex (% male) | 5035 (59%) | 4183 (56%) | 3343 (65%) | 4178 (61%) | 3910 (65%) |
| Peer group deviance | 3,084 | 2,791 | 2,091 | 2,528 | 2,376 |
| Lack of parental monitoring | 2,642 | 2,450 | 1,808 | 2,146 | 2,039 |
| Depressive symptoms | 2,984 | 2,748 | 2,020 | 2,447 | 2,294 |
| Sensation seeking | 2,966 | 2,738 | 2,014 | 2,439 | 2,285 |
| Antisocial behavior | 2,849 | 2,667 | 1,958 | 2,327 | 2,210 |
| Neuroticism | 2,980 | 2,745 | 2,019 | 2,445 | 2,292 |
| Extraversion | 2,980 | 2,745 | 2,019 | 2,443 | 2,291 |
| Openness | 2,979 | 2,745 | 2,019 | 2,442 | 2,290 |
| Agreeableness | 2,977 | 2,744 | 2,019 | 2,442 | 2,291 |
| Conscientiousness | 2,980 | 2,745 | 2,019 | 2,444 | 2,291 |
| Family history of alcohol problems | 3,631 | 3,089 | 2,431 | 3,020 | 2,826 |

| **Table S2. Estimated association (standardized coefficients) linking genetic and psychosocial variables with the intercept and growth of AUDIT alcohol consumption subscale score. Data from The Avon Longitudinal Study of Parents and Children Study 1991-2014.** | | | | |  |
| --- | --- | --- | --- | --- | --- |
|  | **Intercept =4.15** | **Slope 1= 0.45** | **Slope 2=1.04** | **Quadratic growth=**  **-0.18** | |
| **Female** | -0.06 (-0.17, 0.05) | **-1.19 (-1.80, -0.58)** | 0.18 (-0.03, 0.39) | -0.25 (-0.49, -0.01) | |
| **Polygenic score for alcohol consumption** | **0.10 (0.06, 0.14)** | -0.19 (-0.45, 0.08) | 0.06 (-0.03, 0.14) | -0.05 (-0.14, 0.04) | |
| **Family history of alcohol problems** | **0.17 (0.02, 0.32)** | -0.02 (-0.96, 0.93) | 0.20 (-0.10, 0.50) | -0.29 (-0.63, 0.05) | |
| **Lack of parental monitoring** | **0.08 (0.03, 0.13)** | **-0.30 (-0.61, -0.01)** | 0.02 (-0.08, 0.11) | -0.04 (-0.14, 0.07) | |
| **Sensation seeking** | **0.16 (0.11, 0.22)** | -0.15 (-0.51, 0.20) | 0.02 (-0.09, 0.13) | -0.02 (-0.14, 0.09) | |
| **Antisocial behavior** | **0.23 (0.17, 0.29)** | **-0.41 (-0.79, -0.03)** | -0.01 (-0.13, 0.10) | -0.01 (-0.14, 0.12) | |
| **Peer group deviance** | -0.01 (-0.06, 0.05) | -0.11 (-0.47, 0.26) | **-0.11 (-0.22, -0.01)** | **0.13 (0.01, 0.26)** | |
| **Neuroticism** | -0.01 (-0.06, 0.05) | 0.15 (-0.19, 0.49) | 0.02 (-0.08, 0.13) | -0.02 (-0.14, 0.10) | |
| **Extraversion** | **0.12 (0.07, 0.17)** | 0.24 (-0.05, 0.53) | **-0.09 (-0.19, -0.01)** | 0.06 (-0.04, 0.17) | |
| **Openness** | **-0.07 (-0.12, -0.02)** | 0.13 (-0.19, 0.44) | **0.10 (0.003, 0.20)** | -0.10 (-0.22, 0.01) | |
| **Agreeableness** | **0.07 (0.02, 0.12)** | 0.05 (-0.29, 0.38) | -0.02 (-0.13, 0.09) | 0.02 (-0.10, 0.14) | |
| **Conscientiousness** | -0.04 (-0.10, 0.01) | -0.19 (-0.49, 0.11) | -0.03 (-0.12, 0.07) | 0.03 (-0.07, 0.13) | |
| **Depressive symptoms** | -0.01 (-0.07, 0.05) | -0.08 (-0.44, 0.29) | -0.07 (-0.19, 0.04) | 0.07 (-0.06, 0.19) | |
| **Lifetime cigarette consumption** | **0.18 (0.15, 0.22)** | **-0.25 (-0.48, -0.03)** | **-0.19 (-0.27, -0.11)** | **0.16 (0.08, 0.25)** | |
| **RMSEA= 0.041 (90% CI= 0.033 to 0.048); CFI= 0.977; TLI= 0.902** | | | | |  |
| **Slope 1 represents the growth from age 16.5 to age 18. Slope 2 and quadratic growth represent the growth from age 18 to age 23.** | | | | |  |

| **Table S3. Estimated association (Standardized coefficients) linking genetic and psychosocial variables with the intercept and growth of AUDIT alcohol problem subscale score. Data from The Avon Longitudinal Study of Parents and Children Study 1991-2014.** | | | | |
| --- | --- | --- | --- | --- |
|  | **Intercept=1.47** | **Slope 1=0.44** | **Slope 2=0.57** | **Quadratic growth =**  **-0.09** |
| **Female** | **0.28 (0.16, 0.39)** | **-1.48 (-2.06, -0.90)** | -0.22 (-0.50, 0.06) | 0.12 (-0.17, 0.40) |
| **Polygenic score for alcohol problems** | 0.04 (-0.01, 0.09) | 0.20 (-0.06, 0.45) | -0.03 (-0.13, 0.08) | 0.04 (-0.07, 0.15) |
| **Family history of alcohol problems** | **0.38 (0.17, 0.59)** | **-1.33 (-2.20, -0.45)** | **0.63 (0.18, 1.08)** | **-0.59 (-1.04, -0.13)** |
| **Lack of parental monitoring** | **0.10 (0.04, 0.16)** | -0.25 (-0.54, 0.04) | 0.04 (-0.08, 0.16) | -0.04 (-0.16, 0.08) |
| **Sensation seeking** | **0.08 (0.02, 0.14)** | -0.29 (-0.62, 0.03) | 0.06 (-0.07, 0.20) | -0.03 (-0.17, 0.11) |
| **Antisocial behavior** | **0.28 (0.20, 0.35)** | -0.07 (-0.47, 0.32) | -0.10 (-0.29, 0.09) | 0.12 (-0.07, 0.32) |
| **Peer group deviance** | 0.01 (-0.07, 0.08) | 0.37 (0.01, 0.72) | **-0.19 (-0.36, -0.03)** | **0.20 (0.02, 0.37)** |
| **Neuroticism** | 0.00 (-0.06, 0.07) | 0.12 (-0.20, 0.45) | 0.05 (-0.09, 0.19) | -0.06 (-0.20, 0.09) |
| **Extraversion** | **0.07 (0.01, 0.12)** | 0.19 (-0.08, 0.46) | 0.04 (-0.08, 0.17) | -0.08 (-0.21, 0.05) |
| **Openness** | -0.01 (-0.07, 0.04) | -0.03 (-0.33, 0.27) | 0.06 (-0.08, 0.19) | -0.06 (-0.20, 0.08) |
| **Agreeableness** | 0.04 (-0.02, 0.10) | 0.06 (-0.28, 0.39) | 0.01 (-0.13, 0.15) | -0.02 (-0.16, 0.13) |
| **Conscientiousness** | -0.02 (-0.08, 0.04) | -0.16 (-0.45, 0.14) | -0.11 (-0.24, 0.03) | 0.11 (-0.03, 0.25) |
| **Depressive symptoms** | 0.07 (-0.00, 0.14) | -0.06 (-0.41, 0.29) | 0.07 (-0.08, 0.22) | -0.10 (-0.26, 0.06) |
| **Lifetime cigarette consumption** | **0.15 (0.10, 0.19)** | **-0.28 (-0.49, -0.07)** | -0.03 (-0.14, 0.08) | -0.02 (-0.12, 0.09) |
| **RMSEA=0.028 (90% CI= 0.021 to 0.036); CFI=0.982; TLI=0.927** | | | | |
| **Slope 1 represents the growth from age 16.5 to age 18. Slope 2 and quadratic growth represent the growth from age 18 to age 23.** | | | | |

| **Table S4. Estimated prospective associations linking genetic, familial, and psychosocial variables with alcohol consumption measured by AUDIT using Random-Intercept Regression Models. Data from The Avon Longitudinal Study of Parents and Children Study 1991-2014.** | | | | | | |
| --- | --- | --- | --- | --- | --- | --- |
|  |  | Follow-up age | | | | |
|  |  | 16 | 18 | 19 | 21 | 23 |
|  | Variable (n) | β (95% CI) | β (95% CI) | β (95% CI) | β (95% CI) | β (95% CI) |
| Model 1 | Female (7,279) | -0.1 (-0.2, 0.1) | **-0.3 (-0.5, -0.2)** | **-0.4 (-0.6, -0.3)** | **-0.5 (-0.7, -0.4)** | **-0.8 (-0.9, -0.6)** |
|  | Polygenic score for alcohol consumption (7,279) | **0.2 (0.1, 0.3)** | **0.2 (0.1, 0.3)** | **0.3 (0.2, 0.4)** | **0.2 (0.2, 0.3)** | **0.2 (0.1, 0.3)** |
|  | Family history of alcohol problems (5,022) | **0.6 (0.3, 0.9)** | **0.5 (0.1, 0.8)** | **0.8 (0.4, 1.1)** | **0.4 (0.1, 0.7)** | 0.2 (-0.1, 0.5) |
|  | Lack of parental monitoring (3,322) | **0.5 (0.4, 0.6)** | **0.3 (0.2, 0.4)** | **0.4 (0.3, 0.5)** | **0.2 (0.1, 0.3)** | **0.2 (0.0, 0.3)** |
|  | Peer group deviance (4,080) | **0.5 (0.4, 0.6)** | **0.4 (0.3, 0.5)** | **0.2 (0.1, 0.4)** | 0.1 (-0.0, 0.2) | **0.2 (0.1, 0.3)** |
|  | Sensation seeking (3,882) | **0.8 (0.7, 0.9)** | **0.7 (0.6, 0.8)** | **0.7 (0.6, 0.8)** | **0.5 (0.4, 0.6)** | **0.6 (0.5, 0.7)** |
|  | Antisocial behavior (3,636) | **1.0 (0.9, 1.1)** | **0.8 (0.7, 0.9)** | **0.7 (0.5, 0.8)** | **0.4 (0.3, 0.5)** | **0.4 (0.3, 0.5)** |
|  | Agreeableness (3,895) | 0.0 (-0.0, 0.1) | 0.1 (-0.0, 0.2) | 0.1 (-0.0, 0.2) | 0.1 (0.0, 0.2) | 0.1 (-0.0, 0.2) |
|  | Conscientiousness (3,898) | **-0.5 (-0.5, -0.4)** | **-0.4 (-0.5, -0.3)** | **-0.4 (-0.5, -0.3)** | **-0.3 (-0.4, -0.2)** | **-0.3 (-0.4, -0.2)** |
|  | Extraversion (3,897) | **0.6 (0.5, 0.7)** | **0.6 (0.5, 0.6)** | **0.4 (0.3, 0.5)** | **0.3 (0.2, 0.4)** | **0.3 (0.2, 0.4)** |
|  | Neuroticism (3,898) | 0.1 (-0.0, 0.2) | 0.0 (-0.1, 0.1) | 0.0 (-0.1, 0.1) | -0.0 (-0.1, 0.1) | -0.0 (-0.1, 0.1) |
|  | Openness (3,896) | **-0.1 (-0.2, -0.0)** | -0.1 (-0.2, 0.0) | 0.1 (-0.0, 0.2) | 0.1 (-0.0, 0.2) | 0.0 (-0.1, 0.1) |
|  | Depressive symptoms (3,904) | **0.2 (0.1, 0.3)** | **0.2 (0.1, 0.3)** | **0.1 (0.0, 0.2)** | 0.0 (-0.0, 0.1) | 0.0 (-0.1, 0.1) |
|  | Lifetime cigarette consumption (3,587) | **0.7 (0.6, 0.7)** | **0.5 (0.5, 0.6)** | **0.4 (0.3, 0.4)** | **0.2 (0.1, 0.2)** | **0.2 (0.1, 0.3)** |
| Model 2  (n=2,913) | Female | -0.1 (-0.4, 0.1) | **-0.6 (-0.8, -0.3)** | **-0.5 (-0.7, -0.2)** | **-0.4 (-0.7, -0.2)** | **-0.7 (-1.0, -0.4)** |
|  | Polygenic score for alcohol consumption | **0.2 (0.1, 0.3)** | **0.2 (0.1, 0.3)** | **0.2 (0.1, 0.3)** | **0.2 (0.1, 0.3)** | **0.2 (0.1, 0.3)** |
|  | Family history of alcohol problems | **0.4 (0.0, 0.7)** | 0.3 (-0.0, 0.7) | **0.7 (0.3, 1.1)** | **0.4 (0.1, 0.8)** | 0.2 (-0.2, 0.6) |
|  | Lack of parental monitoring | **0.2 (0.1, 0.3)** | 0.1 (-0.0, 0.2) | **0.1 (0.0, 0.3)** | 0.0 (-0.1, 0.2) | 0.0 (-0.1, 0.1) |
|  | Peer group deviance | -0.0 (-0.1, 0.1) | -0.1 (-0.2, 0.1) | -0.1 (-0.3, 0.0) | **-0.2 (-0.3, -0.0)** | -0.0 (-0.1, 0.1) |
|  | Sensation seeking | **0.4 (0.2, 0.5)** | **0.3 (0.2, 0.4)** | **0.4 (0.2, 0.5)** | **0.3 (0.2, 0.4)** | **0.3 (0.2, 0.5)** |
|  | Antisocial behavior | **0.5 (0.4, 0.6)** | **0.4 (0.2, 0.5)** | **0.4 (0.2, 0.5)** | **0.3 (0.1, 0.4)** | **0.3 (0.1, 0.4)** |
|  | Agreeableness | **0.2 (0.0, 0.3)** | **0.2 (0.0, 0.3)** | 0.1 (-0.0, 0.3) | **0.1 (0.0, 0.3)** | **0.2 (0.0, 0.3)** |
|  | Conscientiousness | -0.1 (-0.2, 0.0) | **-0.2 (-0.3, -0.0)** | **-0.2 (-0.3, -0.1)** | **-0.2 (-0.3, -0.0)** | **-0.2 (-0.3, -0.0)** |
|  | Extraversion | **0.3 (0.2, 0.4)** | **0.4 (0.3, 0.5)** | **0.3 (0.1, 0.4)** | **0.2 (0.1, 0.3)** | **0.2 (0.0, 0.3)** |
|  | Neuroticism | -0.0 (-0.1, 0.1) | 0.0 (-0.1, 0.2) | 0.1 (-0.1, 0.2) | 0.1 (-0.1, 0.2) | 0.1 (-0.1, 0.2) |
|  | Openness | **-0.2 (-0.3, -0.0)** | **-0.1 (-0.2, -0.0)** | 0.0 (-0.1, 0.2) | -0.0 (-0.1, 0.1) | -0.0 (-0.2, 0.1) |
|  | Depressive symptoms | -0.0 (-0.2, 0.1) | -0.0 (-0.2, 0.1) | -0.1 (-0.3, 0.0) | -0.1 (-0.3, 0.0) | -0.1 (-0.3, 0.0) |
|  | Lifetime cigarette consumption | **0.4 (0.3, 0.5)** | **0.3 (0.2, 0.4)** | **0.1 (0.0, 0.2)** | 0.0 (-0.0, 0.1) | 0.1 (-0.0, 0.2) |
| Model 1 adjusted for sex (except for the model for sex). The model for polygenic score additionally adjusted for ancestry.  Model 2 adjusted for ancestry and all variables shown in the table. | | | | | | |

| **Table S5. Estimated prospective associations linking genetic, familial, and psychosocial variables with drinking problems measured by AUDIT using Random-Intercept Regression Models. Data from The Avon Longitudinal Study of Parents and Children Study 1991-2014.** | | | | | | |
| --- | --- | --- | --- | --- | --- | --- |
|  |  | Follow-up age | | | | |
|  |  | 16  n=5,068 | 18  n=3,356 | 19  n=4,168 | 21  n=4,175 | 23  n=3,910 |
|  | Variable of interest | β (95% CI) | β (95% CI) | β (95% CI) | β (95% CI) | β (95% CI) |
| Model 1 | Female (7,279) | **0.5 (0.3, 0.7)** | 0.0 (-0.2, 0.2) | -0.1 (-0.3, 0.1) | **-0.4 (-0.6, -0.2)** | **-0.7 (-0.9, -0.5)** |
|  | Polygenic score for alcohol consumption (7,279) | **0.2 (0.1, 0.3)** | **0.2 (0.1, 0.3)** | **0.2 (0.1, 0.3)** | **0.3 (0.2, 0.4)** | **0.3 (0.2, 0.4)** |
|  | Family history of alcohol problems (5,022) | **1.1 (0.7, 1.4)** | **0.5 (0.1, 0.8)** | **1.2 (0.8, 1.6)** | **1.0 (0.7, 1.4)** | **0.8 (0.4, 1.2)** |
|  | Lack of parental monitoring (3,322) | **0.5 (0.4, 0.6)** | **0.4 (0.2, 0.5)** | **0.5 (0.3, 0.6)** | **0.4 (0.3, 0.6)** | **0.3 (0.2, 0.5)** |
|  | Peer group deviance (4,080) | **0.5 (0.4, 0.7)** | **0.6 (0.5, 0.7)** | **0.4 (0.3, 0.5)** | **0.5 (0.4, 0.6)** | **0.5 (0.3, 0.6)** |
|  | Sensation seeking (3,882) | **0.6 (0.5, 0.7)** | **0.5 (0.4, 0.7)** | **0.6 (0.5, 0.7)** | **0.7 (0.6, 0.8)** | **0.7 (0.5, 0.8)** |
|  | Antisocial behavior (3,636) | **1.0 (0.8, 1.1)** | **0.9 (0.8, 1.0)** | **0.8 (0.7, 1.0)** | **0.9 (0.7, 1.0)** | **0.8 (0.7, 1.0)** |
|  | Agreeableness (3,895) | 0.0 (-0.1, 0.2) | -0.0 (-0.1, 0.1) | -0.0 (-0.1, 0.1) | 0.0 (-0.1, 0.1) | -0.1 (-0.2, 0.0) |
|  | Conscientiousness (3,898) | **-0.4 (-0.5, -0.3)** | **-0.4 (-0.5, -0.2)** | **-0.5 (-0.6, -0.4)** | **-0.5 (-0.6, -0.4)** | **-0.4 (-0.5, -0.3)** |
|  | Extraversion (3,897) | **0.4 (0.3, 0.5)** | **0.3 (0.2, 0.4)** | **0.3 (0.2, 0.4)** | **0.3 (0.2, 0.4)** | **0.1 (0.0, 0.2)** |
|  | Neuroticism (3,898) | **0.3 (0.2, 0.4)** | **0.2 (0.1, 0.3)** | **0.2 (0.1, 0.4)** | **0.3 (0.1, 0.4)** | **0.2 (0.0, 0.3)** |
|  | Openness (3,896) | -0.1 (-0.2, 0.0) | -0.0 (-0.2, 0.1) | -0.0 (-0.1, 0.1) | 0.1 (-0.0, 0.2) | 0.0 (-0.1, 0.1) |
|  | Depressive symptoms (3,904) | **0.5 (0.4, 0.6)** | **0.3 (0.2, 0.5)** | **0.4 (0.3, 0.6)** | **0.4 (0.3, 0.6)** | **0.3 (0.2, 0.4)** |
|  | Lifetime cigarette consumption (3,588) | **0.6 (0.6, 0.7)** | **0.4 (0.4, 0.5)** | **0.4 (0.3, 0.5)** | **0.3 (0.3, 0.4)** | **0.2 (0.2, 0.3)** |
| Model 2  (n=2,913) | Female | **0.6 (0.3, 0.9)** | -0.1 (-0.3, 0.2) | -0.3 (-0.6, 0.1) | **-0.5 (-0.8, -0.2)** | **-0.6 (-0.9, -0.3)** |
|  | Polygenic score for alcohol problems | 0.1 (-0.0, 0.2) | **0.2 (0.1, 0.3)** | **0.2 (0.0, 0.3)** | **0.2 (0.1, 0.3)** | **0.2 (0.1, 0.3)** |
|  | Family history of alcohol problems | **0.8 (0.4, 1.2)** | 0.1 (-0.3, 0.5) | **1.0 (0.6, 1.5)** | **0.7 (0.3, 1.2)** | **0.7 (0.3, 1.2)** |
|  | Lack of parental monitoring | **0.2 (0.1, 0.3)** | 0.1 (-0.0, 0.2) | 0.1 (-0.0, 0.3) | 0.1 (-0.0, 0.3) | 0.1 (-0.0, 0.3) |
|  | Peer group deviance | 0.0 (-0.1, 0.2) | **0.2 (0.0, 0.3)** | 0.0 (-0.2, 0.2) | -0.0 (-0.2, 0.2) | 0.1 (-0.0, 0.3) |
|  | Sensation seeking | **0.2 (0.0, 0.3)** | 0.0 (-0.1, 0.2) | 0.1 (-0.1, 0.3) | **0.2 (0.0, 0.3)** | **0.2 (0.1, 0.4)** |
|  | Antisocial behavior | **0.6 (0.5, 0.8)** | **0.6 (0.5, 0.8)** | **0.4 (0.2, 0.6)** | **0.5 (0.4, 0.7)** | **0.6 (0.4, 0.8)** |
|  | Agreeableness | 0.1 (-0.0, 0.2) | 0.1 (-0.0, 0.3) | 0.1 (-0.1, 0.3) | **0.2 (0.0, 0.3)** | 0.1 (-0.0, 0.3) |
|  | Conscientiousness | -0.0 (-0.2, 0.1) | -0.1 (-0.2, 0.0) | **-0.2 (-0.4, -0.0)** | **-0.2 (-0.4, -0.1)** | **-0.2 (-0.3, -0.0)** |
|  | Extraversion | **0.2 (0.0, 0.3)** | **0.2 (0.1, 0.4)** | **0.3 (0.1, 0.4)** | **0.2 (0.1, 0.4)** | 0.1 (-0.1, 0.2) |
|  | Neuroticism | 0.0 (-0.1, 0.2) | 0.1 (-0.1, 0.2) | 0.1 (-0.1, 0.2) | **0.2 (0.0, 0.3)** | 0.1 (-0.1, 0.2) |
|  | Openness | -0.0 (-0.2, 0.1) | -0.0 (-0.2, 0.1) | 0.0 (-0.1, 0.2) | -0.0 (-0.2, 0.1) | -0.0 (-0.2, 0.1) |
|  | Depressive symptoms | 0.1 (-0.0, 0.3) | 0.1 (-0.1, 0.2) | **0.2 (0.1, 0.4)** | 0.1 (-0.1, 0.3) | 0.0 (-0.1, 0.2) |
|  | Lifetime cigarette consumption | **0.3 (0.2, 0.4)** | **0.2 (0.1, 0.3)** | **0.2 (0.1, 0.3)** | 0.1 (-0.0, 0.2) | -0.0 (-0.1, 0.1) |
| Model 1 adjusted for sex (except for the model for sex). The model for polygenic score additionally adjusted for ancestry.  Model 2 adjusted for ancestry and all variables shown in the table. | | | | | | |

Based on results from random-intercept models (Table S4, Model 1), almost all variables under study predicted alcohol consumption at each wave of assessment. Prominent psychosocial predictors include indicators of externalizing problems such as antisocial behaviors and sensation seeking, and cigarette consumption, for which higher levels predicted greater alcohol consumption. Other variables inversely predicted alcohol consumption, including conscientiousness and openness. The strength of these predictions tended to decrease as individuals grew from adolescents to young adults. The PRS for alcohol consumption was consistently associated with alcohol consumption over time. A family history of alcohol problems also predicted alcohol consumption, and the strength of the prediction remained relatively stable over time. Females consumed less alcohol throughout the study period compared to males, and the difference tended to grow over time. Estimates attenuated after adjusting for all variables under study (Table S3, Model 2).

With respect to the AUDIT composite score for alcohol problems, we observed robust associations for all variables studied except for openness (Table S5, Model 1). Unlike the decreasing strength pattern observed for alcohol consumption, the strength of association remained relatively stable. We observed a reversal of male-female difference in the level of drinking problems. That is, adolescent girls had higher levels of problematic drinking at 16; the sex difference became null at 18 and 19; from age 21 on, a male excess was observed, and it grew larger during young adulthood. Depressive symptoms predicted alcohol consumption at younger age, but the association diminished at age 21. In contrast, depressive symptoms were associated with problematic drinking throughout the study period.

**References**

**Angold, A., Costello, E. J. & Messer, S. C.** (1995). Development of a short questionnaire for use in epidemiological studies of depression in children and adolescents. *International Journal of Methods in Psychiatric Research* **5**, 237-249.

**Arnett, J.** (1994). Sensation Seeking - a New Conceptualization and a New Scale. *Personality and Individual Differences* **16**, 289-296.

**Babor, T. & Grant, M.** (1989). From clinical research to secondary prevention: International collaboration in the development of the Alcohol Use Disorders Identification Test (AUDIT). *Alcohol Health and Research World* **13**, 371-374.

**Ehrhart, K. H., Roesch, S. C., Ehrhart, M. G. & Kilian, B.** (2008). A test of the factor structure equivalence of the 50-item IPIP Five-factor model measure across gender and ethnic groups. *Journal of Personality Assessment* **90**, 507-16.

**Goodman, R., Ford, T., Simmons, H., Gatward, R. & Meltzer, H.** (2000). Using the Strengths and Difficulties Questionnaire (SDQ) to screen for child psychiatric disorders in a community sample. *British Journal of Psychiatry* **177**, 534-9.
